# Sweet Spot of Intermolecular Coupling in Crystalline Rubrene: Intermolecular Separation to Minimize Singlet Fission and Retain Triplet–Triplet Annihilation

**DOI:** 10.1021/acs.jpcc.2c04572

**Published:** 2022-08-30

**Authors:** P. Baronas, G. Kreiza, L. Naimovičius, E. Radiunas, K. Kazlauskas, E. Orentas, S. Juršėnas

**Affiliations:** † Institute of Photonics and Nanotechnology, Vilnius University, Sauletekio 3, LT-10257 Vilnius, Lithuania; ‡ Institute of Chemistry, Faculty of Chemistry and Geosciences, Vilnius University, Naugarduko 24, LT-03225 Vilnius, Lithuania

## Abstract

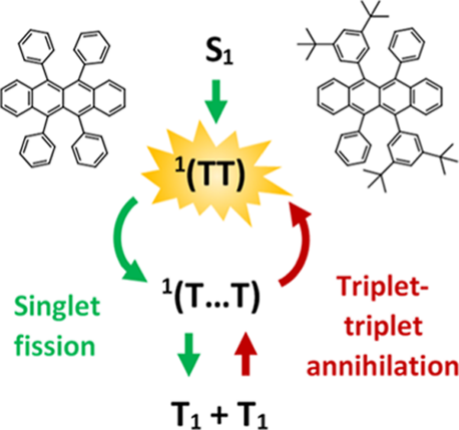

Singlet fission is detrimental to NIR-to-vis photon upconversion in the solid rubrene (Rub) films, as it diminishes photoluminescence efficiency. Previous studies have shown that thermally activated triplet energy transport drives singlet fission with nearly 100% efficiency in closely packed Rub crystals. Here, we examine triplet separation and recombination as a function of intermolecular distance in the crystalline films of Rub and the *t*-butyl substituted rubrene (*t*BRub) derivative. The increased intermolecular distance and altered molecular packing in *t*BRub films cause suppressed singlet dissociation into free triplets due to slower triplet energy transport. It was found that the formation of correlated triplet pairs ^1^(TT) and partial triplet separation ^1^(T···T) occurs in both Rub and *t*BRub films despite differences in intermolecular coupling. Under weak intermolecular coupling as in *t*BRub, geminate triplet annihilation of ^1^(T···T) outcompetes dissociation into free triplets, resulting in emission from the ^1^(TT) state. Essentially, increasing intermolecular distance up to a certain point (a sweet spot) is a good strategy for suppressing singlet fission and retaining triplet–triplet annihilation properties.

## Introduction

Rubrene (Rub) is a simple molecule that in the solid state transforms into a multifunctional material enabling applications in near-infrared photon upconversion (NIR-UC),^1,2^ organic light-emitting diodes (OLEDs),^3,4^ and organic transistors.^5,6^ Features such as fast charge transport,^7^ long-range triplet energy transport,^8−10^ efficient singlet fission (SF),^11,12^ and triplet–triplet annihilation (TTA)^13^ are related to the unique electronic structure of Rub molecules and their specific arrangement in crystals. In Rub singlet energy is nearly twice as large as triplet energy (S_1_ ∼ 2 × T_1_), allowing to double photon energy gain in the TTA emitter, yet also resulting in ultrafast SF. The SF and TTA processes in Rub crystals and films are well reviewed in the recent papers by Bossanyi et al.^13,14^ It is now widely accepted that SF is a three-step process^15^
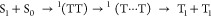
1where the initial step forms a correlated triplet state ^1^(TT) later evolving into the ^1^(T···T) state as it losses electronic coherence between triplets but retains the spin-0 character and subsequent spin decoherence results in full dissociation into free triplets.^16,17^ The efficiency of the SF process strongly depends on the intermolecular coupling dictated by intermolecular distance, that is, an overlap of π-orbitals between neighboring molecules and relative intermolecular orientation.^18^ Many reports have shown that the SF rate is mostly limited by triplet energy transport that allows dissociating the triplet pair.^19−21^ Therefore, weaker intermolecular coupling and slower triplet energy transport are likely to result in a more efficient reverse process of triplet fusion that determines the overall rate of TTA. Moreover, it has been suggested that weaker triplet exchange coupling depending on the degree of wavefunction overlap is beneficial for enhanced spin statistical factor in the TTA process.^13^

Despite being one of the best-performing NIR-to-vis TTA emitters, Rub has a major drawback related to crystallization-induced SF, which significantly reduces photoluminescence quantum yield (PLQY).^2,22−24^ Therefore, Rub must be modified so as to attain optimum intermolecular coupling in the solid state for weak SF and moderate TTA. Reduction of intermolecular coupling could be achieved by randomly orienting Rub molecules in amorphous films^2^ or by increasing their structural complexity.^12,23^ It was shown that with increasing molecular separation, TTA dominates over SF in Rub-based OLEDs doped with various concentrations of the mCP spacer.^25^ However, the introduction of the bulky side-moieties into the Rub molecule was demonstrated to completely suppress SF as well as TTA,^24^ indicating the necessity of optimal electronic coupling (a sweet-spot) for the formation of correlated triplet pairs. Besides SF, other factors such as triplet energy transfer to low-lying defects, for example, Rub peroxide,^26−28^ have been found to deteriorate PLQY of crystalline Rub, thus limiting the NIR-UC efficiency.^14,29^ Importantly, because the SF rate in crystalline Rub exhibits strong temperature activated behavior,^30,31^ this can be detrimental to NIR-UC applications aiming for stable operation.

In our previous work, we demonstrated that Rub modified with aliphatic *t*-butyl groups (*t*BRub) showed 4 times higher NIR-UC efficiency (up to 0.3%) compared to analogous Rub films.^23^ This was attributed to the increased intermolecular distance that suppresses the SF rate.^24^ In contrast, measurements in NIR-UC solution, where SF is negligible, showed at least three times higher efficiency in Rub than in *t*BRub, due to a higher TTA spin statistical factor (*f*) of 15.5 versus 5.3%, respectively.^22^ This indicates that losses due to SF in Rub films are larger than the reduction of TTA probability in *t*BRub, and while both phenomena depend on intermolecular coupling, the underlying mechanism could be different.

In this work, we aim to show that reduced intermolecular coupling suppresses the SF rate more than the TTA rate. To this end, we employed ultrafast time-resolved PL (TRPL) and transient absorption (TA) techniques to probe the dynamics of intermediate triplet pair states ^1^(TT) and ^1^(T···T) in Rub and *t*BRub polycrystalline films. Here, the SF rate was identified via triplet separation within picoseconds in TA, while geminate TTA was monitored via delayed ^1^(TT) emission within nanoseconds after excitation. The intermolecular packing properties in both crystals were determined by X-ray diffraction (XRD) and compared to the signatures of excitonic coupling emerging in absorption and emission spectra. Temperature-dependent measurements enabled us to extract activation energies for SF and geminate TTA.

## Methods

### Polycrystalline Film Preparation and Characterization

Sublimation grade Rub was purchased from TCI. The synthesis of *t*BRub was published elsewhere.^22^*t*BRub was purified by vacuum sublimation prior to use in the experiments. Polycrystalline films for optical experiments were prepared by heating powder up to the melting point between two glass slides and slowly allowing it to cool down. Single crystals of small dimensions (100 μm × 100 μm) for XRD analysis were grown by heating the initial powder in a closed vial at similar conditions to polycrystalline samples. Suitable crystals were mounted and analyzed on an XtaLab Synergy diffractometer with a HyPix-6000HE hybrid photon counting detector and a PhotonJet microfocus X-ray source (Cu Kα, λ = 1.54184). The structures were solved by intrinsic phasing with the ShelXT^32^ program and refined with the ShelXL^33^ package using least squares minimization implemented in the Olex2 graphical interface.^34^ Obtained structures were deposited to the Cambridge Crystallographic Data Centre and can be accessed free of charge (**CCDC** deposition numbers: **2170150** and **2169522**). Morphology of the investigated polycrystalline films was characterized by using a powder XRD measurement regime employing the same experimental setup. Samples for measurement were obtained from Rub and tBRub films which were peeled off from the substrate.

### Optical Spectroscopy

Absorption spectra were recorded on a UV–vis–NIR spectrophotometer LAMBDA 950 (PerkinElmer). Temperature-dependent nanosecond TRPL spectra were recorded in nitrogen cryostat detecting PL with a streak scope C10627 detector (Hamamatsu) exciting samples with 460 nm pulses (repetition rate 10 kHz, pulse duration 190 fs) using laser system Pharos-SP coupled to parametric amplifier Orpheus (Light Conversion). Temperature-dependent microsecond TRPL spectra were recorded in temperature-controlled closed-cycle helium cryostat detecting PL with an iCCD camera (New iStar DH340T, Andor) exciting the samples with 460 nm emission of a tunable-wavelength optical amplifier (Ekspla) pumped by a nanosecond Nd^3+^:YAG laser(pulse duration—5 ns, repetition rate—1 kHz). Femtosecond TA measurements were carried out using a Harpia spectrometer pumped with 485 nm pulses from a Pharos-SP laser and an Orpheus parametric amplifier system (Light Conversion). The probe source was white light continuum pulses generated by focusing the 1030 nm in purified water flowing inside a quartz cuvette coupled to a home-built flow system. Temperature-dependent TA measurements were performed by mounting the samples in a nitrogen cryostat. Global analysis of TA was performed by data analysis software “CarpetView” (Light Conversion). A sequential model was used to separate spectral features of two states and assign corresponding decay lifetimes.^35,36^

## Results and Discussion

**Figure 1:**
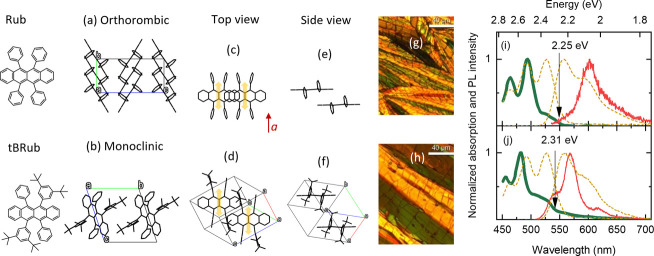
XRD crystal structures of Rub and tBRub (a–f). For clarity closest neighbor molecules in the crystal are shown in (c–f). Arrows in (c–d) indicate singlet transition dipole moments in Rub-based molecules. Micrographs of polycrystalline films recorded with crossed polarizations (g–h). Room temperature absorption (thick line) and PL (thin line) spectra of polycrystalline films (i–j). Corresponding absorption and emission spectra of toluene solutions (10^–5^ M) are shown by a dashed line. The energy gap is indicated.

Polycrystalline films of Rub and *t*BRub were prepared via melt processing. Figure 1a–f shows molecular packing in crystals of Rub and *t*BRub determined by XRD measurements of single crystals grown under similar conditions. Powder XRD measurements of polycrystalline films (see Figure S1 in the Supporting Information) suggest a good match between molecular packing in investigated thin films and single crystals. Crystalline domain size in Rub and *t*BRub polycrystalline films was in the range of 1–10 μm (Figure 1g,h). Rub polycrystalline films were dominated by orthorhombic molecular packing, which is the most stable Rub polymorph.^37^ The addition of tertbutyls in *t*BRub resulted in a dominant monoclinic crystal form. The differences between the two forms are evident from the top and side views, which show the reduced overlap of π-systems in the *t*BRub compared to Rub. The addition of tertbutyls in *t*BRub increased intermolecular separation in crystals, which must affect the excitonic properties as well. The intermolecular separation in the *b* direction was 7.160 Å in Rub crystal and 10.614 Å in *t*BRub crystal (see Table S1 in the Supporting Information). An even larger difference was recorded in distance between π–π planes of the tetracene core, which was approximately 3.7 Å in Rub crystals and 6.9 Å in *t*BRub crystals. It is known that charge transfer (CT) excitons form at close intermolecular distances (∼3.5–4 Å) due to the overlap of frontier molecular orbitals.^38^ Therefore, orthorhombic Rub suggests strong mixing between CT excitons, where charges are located on neighboring molecules, and Frenkel excitons created by dipole–dipole interaction between molecules. In contrast, large intermolecular separation in monoclinic *t*BRub crystals should result in the formation of pure Frenkel excitons. In Rub transition dipole moment for singlet S_0_–S_1_ transition is polarized along the short molecular axis of tetracene core.^39^ The side-by-side stacking of transition dipole moments (Figure 1c,d) implies the formation of H-type Frenkel excitons in both Rub and *t*BRub.

The differences in molecular packing and intermolecular coupling are reflected in the absorption and emission spectra of Rub and *t*BRub polycrystalline films shown in Figure 1i,j. Singlet energy gap S_0_–S_1_ for crystalline Rub was determined to be approximately 2.25 eV from the onset of absorption spectra. It was in agreement with the values recorded for orthorhombic Rub crystals.^11,39−42^ Additionally, S_0_–S_1_ energy gap of orthorhombic Rub crystal is very close to S_0_–S_1_ of Rub monomer in a toluene environment. The negligible spectral shift in Rub crystals indicates that the effects of excitonic coupling on energy are canceled out possibly due to similar H-type Frenkel and J-type CT coupling. This is in agreement with negligible Davydov splitting observed in orthorhombic Rub.^41^ In contrast, different molecular packing of monoclinic *t*BRub crystal results in approximately 60 meV higher S_0_–S_1_ energy gap of 2.31 eV compared to Rub crystals. Larger intermolecular separation and weaker π-interaction imply weaker CT coupling in monoclinic *t*BRub. Here, dipole–dipole interactions are not as sensitive to the changes in intermolecular distance, and therefore, spectral blueshift may indicate dominant H-type Frenkel exciton. Similarly, the monoclinic polymorph of Rub crystals has been theoretically and experimentally shown to have an even larger energy gap of 2.36 eV.^37,42^ Significant blueshift of absorption spectra in monoclinic Rub was induced by the change of relative molecular orientation (similar to the one displayed in Figure 1d) rather than increased intermolecular distance. Therefore, it is difficult to predict the strength and type of CT excitonic coupling in *t*BRub crystals only by interpreting the increased distance between π-planes of neighboring molecules. It is important to note, that CT excitons mediate SF, and coupling strength determines the reaction rate.^43^ Furthermore, 60 meV higher S_0_–S_1_ energy gap of *t*BRub crystal compared to Rub is likely to influence temperature dependence of SF dynamics.

**Figure 2:**
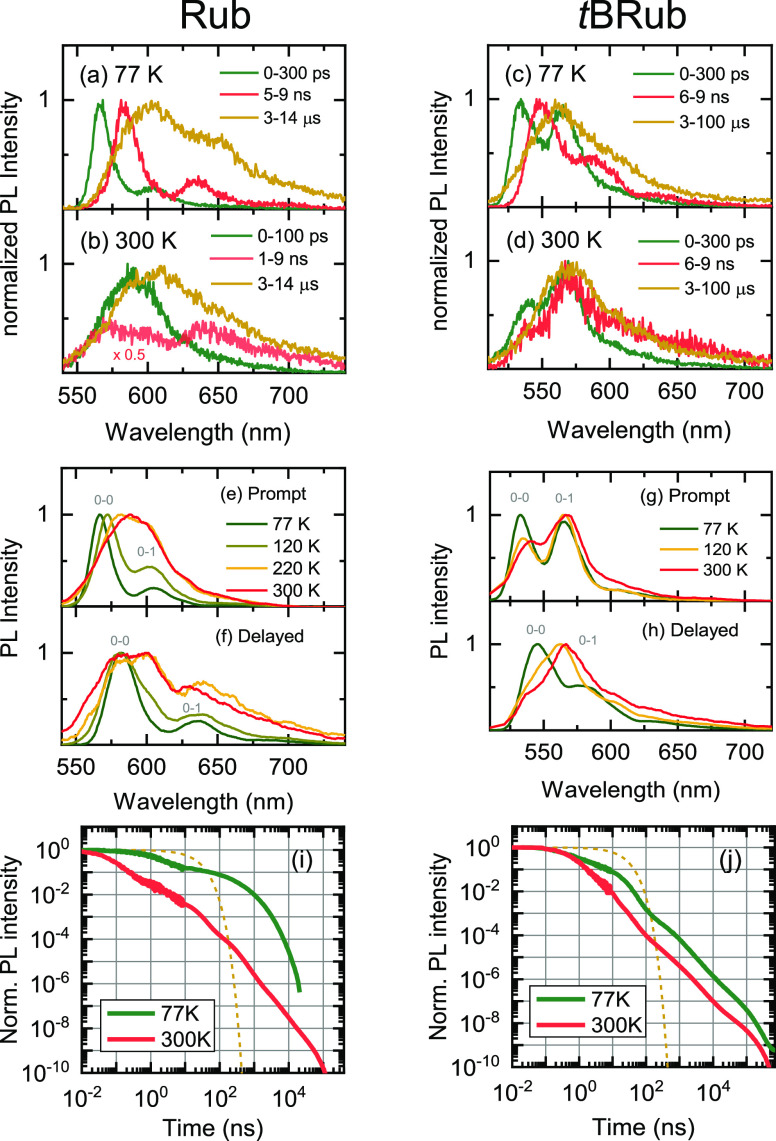
Temperature-dependent time-resolved photoluminescence of Rub and *t*BRub polycrystalline films. (a–d) PL spectra at significant delay times measured at 77 and 300 K temperatures. (e–h) Decay-associated spectra of prompt and delayed emission obtained from global analysis of TRPL data. (i,j) Normalized spectrally integrated PL transients at 77 and 300 K. Transients up to 10 ns were obtained with a streak camera setup, and transients from 10 ns were measured with a gated ICCD camera. Single-exponential decay curve (dashed line) with a 19 ns lifetime serves as a reference for the decay of the Rub monomer in solution.

To understand the complex PL emission in both Rub and *t*BRub polycrystalline films, we performed TRPL measurements as a function of temperature (Figure 2). Spectral evolution indicated the multiple decay components associated with different emissive species in both films. In Rub films at 77 K, prompt emission peaking at 566 nm during the first few hundred picoseconds and delayed red-shifted emission peaking at 583 nm within nanoseconds could be resolved (Figure 2a). Both bands shared almost identical spectral forms, yet were separated by 60 meV. These low-temperature emission components were previously reported in Rub single crystals.^26−28,39^ Microsecond-delayed emission spectrum contained delayed emission component along with a new 650 nm band previously assigned to oxygen-related defects in crystalline Rub.^26−28^ At room temperature, PLQY of Rub films was significantly suppressed due to picosecond SF (Figure 2b), which made it difficult to resolve all the emission components. The prompt emission spectrum was significantly broader with no vibronic features, however, the relative defect emission intensity remained similar to the 77 K spectrum. In the case of *t*BRub polycrystalline films at 77 K, nanosecond prompt and delayed components could be also resolved. Figure 2c shows prompt emission within a few hundred picoseconds peaking at 531 nm and nanosecond-delayed emission redshifted to 547 nm. Interestingly, delayed nanosecond emission in *t*BRub was shifted by 60 meV relatively to prompt emission, which was identical to the redshift observed in Rub films. Microsecond-delayed emission peaking at 563 nm resembles spectral superposition of prompt and delayed components together with a weak emission at 600–650 nm, which may indicate defect formation. Unlike Rub films, *t*BRub exhibited significantly weaker temperature dependence of PLQY due to suppressed SF. Therefore, spectral signatures of prompt and delayed emission were still visible at room temperature (Figure 2d). Furthermore, microsecond-delayed emission of *t*BRub films shows suppressed emission from the peroxide defect band, which could indicate the increased resistance to oxidation under identical crystal growth conditions.

To extract spectra and lifetimes of two emitting components in the picosecond to nanosecond TRPL data, we performed a global analysis using a sequential model (for more details see Section S2 in the Supporting Information). The decay-associated spectra of Rub and *t*BRub polycrystalline films as a function of temperature are presented in Figure 2e–h. The corresponding decay lifetimes of prompt (τ_p_) and delayed (τ_d_) components are shown in Table 1. Interestingly, Figure 2e,f shows that both prompt and delayed emission of polycrystalline Rub films exhibit a strongly enhanced 0–0 vibronic band at low temperatures compared to the Rub monomer emission. This is a clear indication of superradiance occurring due to the collective enhancement of transition dipoles in *J*-aggregates.^38,44^ The 0–0/0–1 intensity ratio is reduced at elevated temperatures due to exciton–phonon interaction that suppresses exciton delocalization. Mitrofanov et al. performed emission polarization analysis of Rub crystals revealing that 565 nm (2.19 eV) emission originated from the M-polarized transition.^27^ The M transition coincides with the *a*-axis in the orthorhombic Rub crystal and the direction of molecular dipole moments within the crystal (Figure 1c). However, strong J-type excitonic coupling may originate from CT interactions in Rub crystals. Nevertheless, the origin of red-shifted delayed emission (0–0 band at 583 nm) appearing in Rub crystals was not yet discussed in detail.^26,28^ Similar prompt and delayed emission components could be resolved for *t*BRub polycrystalline films (Figure 2g,h). Here, a considerably lower 0–0/0–1 intensity ratio at low temperatures reflected weaker intermolecular coupling in *t*BRub crystals. This ratio in crystals at 77 K was lower than in solution, indicating weak H-type dipole–dipole coupling. It must be noted that exciton delocalization with an admixture of CT in the exciton wavefunction was suggested to be a driving force for efficient SF.^45,46^

The nanosecond-delayed emission observed in Rub and *t*BRub polycrystalline films (Figure 2f,h) may potentially originate from ^1^(TT) states due to the geminate triplet pair recombination. The emission from otherwise optically dark singlet state in various oligoacene crystals showing SF was recently explained via the Herzberg–Teller intensity borrowing mechanism.^47,48^ Here, the red-shifted emission appears as vibrational modes and induces the mixing of the dark ^1^(TT) state with S_1_ states borrowing its oscillator strength. Meanwhile, the decay lifetime of the ^1^(TT) emission is limited by geminate recombination of the ^1^(T···T) state. Similar delayed and red-shifted emission features were found in tetracene^49^ and TIPS-tetracene,^19^ which are closely related to Rub. Furthermore, rapid geminate recombination was found to dominate SF dynamics in tetracene polycrystalline films at low temperatures due to suppressed triplet energy transport inhibiting full triplet separation.^50^ The delayed emission from ^1^(TT) states also persists in the nanosecond to microsecond range as a result of TTA from dissociated triplets, that can annihilate geminately (same triplet pair generated via SF) or nongeminately (triplets from different SF events).

TTA dynamics in Rub and *t*BRub polycrystalline films can be evidenced from picosecond-to-microsecond-delayed PL transients as a function of temperature. Figure 2i shows that in Rub films at 300 K, a significant singlet population is converted to triplets within the first hundred picoseconds, where the following power-law decay of delayed ^1^(TT) emission is produced by geminate and nongeminate TTA. This is in agreement with power-law-delayed PL observed in Rub crystals and amorphous films.^51−53^ It was shown that geminate recombination also persists in microsecond delay times as separated triplets travel in different crystallographic directions of highly anisotropic Rub crystals to recombine again resulting in the power law dynamics of delayed emission.^54^ At 77 K, the SF in Rub films is significantly suppressed resulting in the long decay of prompt emission; however, TTA can still be evidenced by substantial delayed PL signal microseconds after excitation. The initial decay of PL transients in Rub films is significantly faster than the 19 ns lifetime of the Rub monomer, indicating dissociation of triplet pairs even at low temperatures (Figure 2i). While slower decay of the microsecond-delayed PL in Rub films at 77 K indicates substantially slower triplet energy transport at low temperatures.^46^ In contrast, the *t*BRub polycrystalline film showed considerably weaker temperature dependence of prompt and delayed PL dynamics (Figure 2j). The decay lifetime of prompt emission showed inverse temperature dependence, indicating enhancement of SF rate at low temperatures (Table 1). This can be related to an S_1_ energy excess with respect to 2 × T_1_ that should result in a lower energy barrier for triplet dissociation compared to Rub films. Meanwhile, the longer lifetime and relative emission intensity of nanosecond-delayed emission at low temperatures suggest more efficient geminate recombination. Nevertheless, microsecond-delayed emission indicated that a significant triplet population is still generated via SF leading to TTA after triplets have migrated out of their initial position. Interestingly, the slope of power-law decay of delayed emission in the 10 ns to 100 μs time range was weakly temperature dependent suggesting that triplet energy transport in *t*BRub films also has a lower thermal activation barrier than in Rub films. Moreover, the slope of delayed PL decay in the 10 ns to 10 μs time range in *t*BRub films was similar to Rub films at 300 K (see Figure S4 in the Supporting Information). This suggests that the rate of TTA in both Rub and *t*BRub films is on the same order of magnitude at room temperature.

**Table 1:** Lifetimes Obtained from Global Analysis of TA and TRPL Data of Rub and *t*BRub Polycrystalline Films^a^

	Rub			*t*BRub		
	TA	TRPL		TA	TRPL	
*T* (K)	τ (ps)	τ_p_ (ps)	τ_d_ (ps)	τ (ps)	τ_p_ (ps)	τ_d_ (ps)
77	407	900	2050	133	180	2000
150	210	260	1250	124		
220	72	94	847	205	210	1200
300	19	18	1240	265	250	1000

a Prompt and delayed component lifetimes in the picosecond to nanosecond timescale are indicated as τ_p_ and τ_d_, respectively.

To estimate the differences in SF rate in Rub and *t*Brub polycrystalline films, we performed TA measurement as it allows us to directly observe both singlet and triplet states.^11,30,55^ In TA measurements, the possibility of separately observing spectral signatures of ^1^(TT) state is debated due to overlap with other signals.^30,48^ In Rub crystals, S_1_ and ^1^(TT) states were found to be in near resonance resulting in the subpicosecond time-domain transitions between these states; the subsequent picosecond dynamics of TA signal was attributed to spatial separation of the triplets. Bera et al. showed that triplets in Rub crystals separate within 10 ps can be directly measured using ultrafast Raman spectroscopy.^56,57^ The ability to observe triplet signal in TA allows to evaluate the efficiency of triplet separation ^1^(TT) → ^1^(T···T) and to determine whether it is limited by geminate recombination ^1^(T···T) → ^1^(TT).

**Figure 3:**
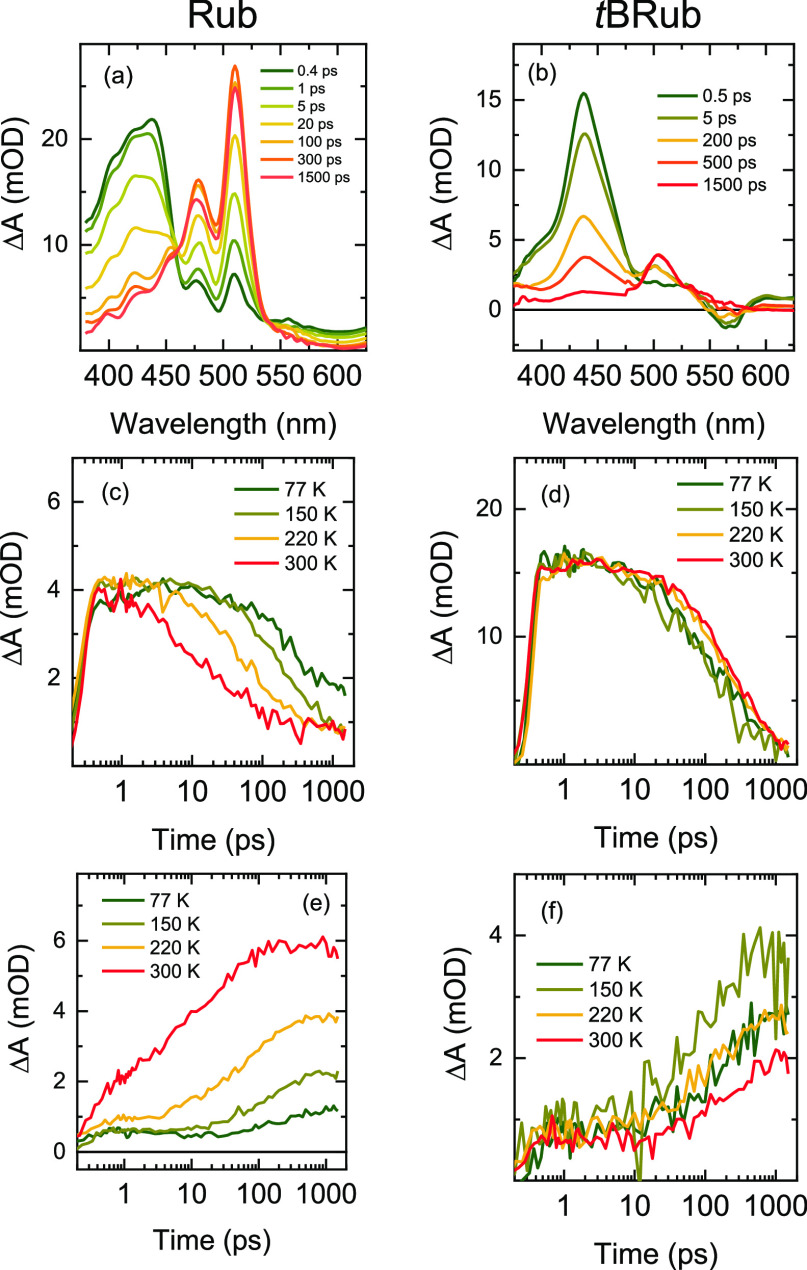
Temperature-dependent TA spectra of Rub and *t*BRub polycrystalline films. (a,b) TA spectra at significant delay times recorded at 300 K. Transients of Rub polycrystalline films recorded at 410 (c) and 515 nm (e) and *t*BRub polycrystalline films recorded at 430 (d) and 505 nm (f).

Figure 3a shows typical SF dynamics of crystalline Rub films.^11^ Excited state absorption (ESA) signal in the 390–450 nm range is associated with singlet S_1_–S_3_ transition decaying within the tens of picoseconds to generate the triplet T_1_–T_3_ ESA signal at 515 nm.^58^ High efficiency of SF in Rub films at 300 K is evidenced by increasing intensity of the ground state bleach signal at 493 nm (corresponding to the absorption peak) at later delay times, which shows that decay of one singlet excitation results in bleaching of more than one molecule after SF. At lower temperatures signal in Rub films decayed significantly slower (Figure 3c), followed by slower rise time and reduced intensity of triplet signal (Figure 3e), thus confirming temperature-activated SF. Global analysis of TA data was used to extract decay lifetimes at each temperature setting (for more details see Section S4 in the Supporting Information). Rub films at 300 K exhibited the dominant 19 ps decay component (Table 1), which was similar to values found in the literature for orthorhombic Rub.^11,30,31^ At 77 K 20-times longer lifetime of 407 ps was recorded, simultaneously, the maximum intensity of triplet ESA was reduced 6-fold. It must be noted, that decay lifetime in crystalline Rub further increases upon lowering the temperature reaching 1 ns around 50 K.^31^ However, even at low temperatures SF in crystalline Rub is not completely suppressed and populates triplets via an ultrafast coherent channel that is evidenced by triplet ESA feature at early delay times.^30^

Despite significantly suppressed triplet separation at low temperatures in Rub films, rapid signal decay suggests that triplet separation S_1_ → ^1^(TT) → ^1^(T···T) remains a dominant pathway. Considering the 15.2 ns singlet lifetime of amorphous Rub,^58^ subnanosecond singlet decay in polycrystalline Rub films at low temperatures indicates the dominant rapid nonradiative pathway associated with SF. Another relevant nonradiative pathway is energy transfer to oxygen defects, however, if this was a significant channel, it would be reflected in dominant defect emission at 650 nm nanoseconds after excitation (see Figure 2a). Alternatively, the shortening of singlet decay lifetime could also be induced by coherent enhancement of the radiative rate due to J-type excitonic coupling, the effect should be limited to 5–8 times according to the enhancement of the 0–0/0–1 emission ratio (Figure 2e). Coherent enhancement at low temperatures also results in more rapid singlet diffusion to defects that would consume the excited population. However, the initial excited-state losses in Rub films at low temperature are converted to delayed emission in the nanosecond to microsecond range. Here, previously discussed ^1^(TT) emission is an indication of excitation recycling via geminate recombination of ^1^(T···T) states.

TA spectra of *t*BRub polycrystalline films exhibited similar singlet and triplet ESA features to those of Rub films; however, the decay of singlet ESA at 430 nm was associated with the rise of significantly weaker triplet ESA signal at 510 nm (Figure 3b). 5-fold lower relative triplet ESA intensity compared to singlet ESA intensity already indicates that triplet generation via SF is suppressed in *t*BRub compared to Rub crystals. This agrees with increased PLQY in *t*BRub films.^24^ Unlike in Rub films, the decay rate in *t*BRub films showed weak temperature dependence (Figure 3d). Similarly, triplet signal intensity did not change significantly with temperature (Figure 3f). Nevertheless, the subnanosecond decay of *t*BRub films throughout the temperature range (see Table 1) was substantially faster than the slow radiative rate (0.05 ns^–1^) of the *t*BRub monomer. This shows that initial excitation decay is most likely dominated by triplet separation S_1_ → ^1^(TT)→ ^1^(T···T).

Previous works have suggested that in both endothermic and exothermic SF systems, triplet pair formation S_1_ → ^1^(TT) occurs on subpicosecond timescales and subsequent dissociation of triplet pair is governed by slower thermally activated triplet energy transfer.^19,21,59^ S_1_ and ^1^(TT) were found to be nearly resonant in Rub crystals,^21^ which should result in dynamic equilibrium between them. Then, the decay rate of the initial excited population is governed by the triplet separation rate determined by the energy barrier for triplet hopping as well as intermolecular coupling strength. Therefore, the subnanosecond decay lifetimes of prompt PL component and TA signal observed for both Rub and *t*BRub films (see Table 1) correspond to the ^1^(TT) → ^1^(T···T) process. In Rub films, this triplet pair separation is an endothermic process and requires thermal energy as evidenced by temperature-activated SF dynamics. Meanwhile, weak temperature dependence in *t*BRub implies that triplet separation does not require thermal energy, which agrees well with higher S_1_ energy in *t*BRub crystals. Surprisingly, at a low temperature limit where thermal energy is insufficient to drive rapid triplet separation in Rub films, the ^1^(TT) → ^1^(T···T) process is faster in tBRub films. This may be related to the highly symmetric molecular packing of orthorhombic Rub crystals that necessitates thermal excitation of symmetry breaking modes to activate electronic coupling for triplet separation.^30^ Lower symmetry of *t*BRub crystals may not require thermal activation of vibrational modes to induce triplet separation.

**Figure 4:**
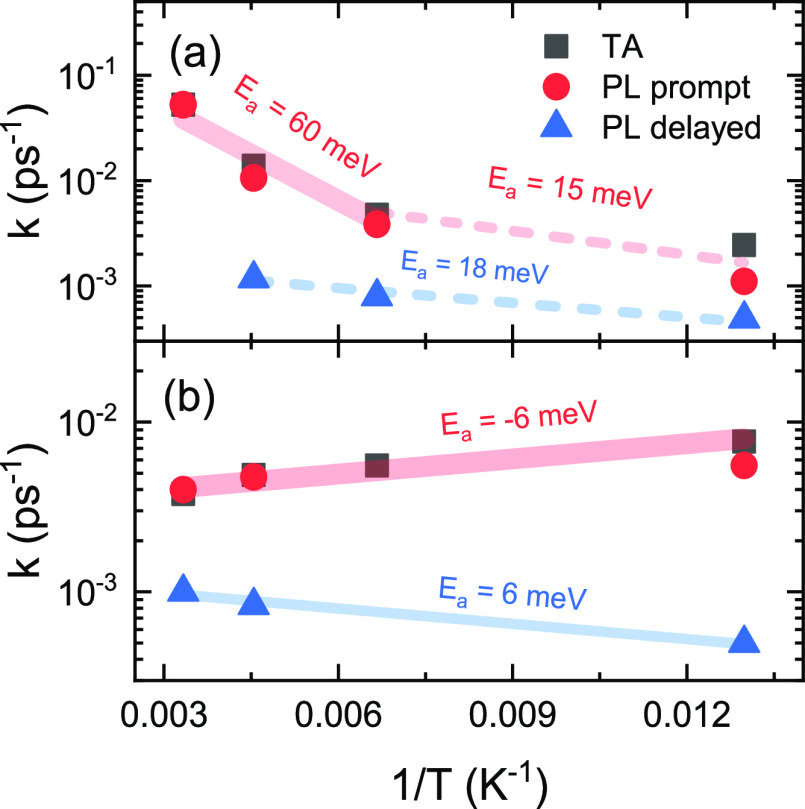
Arrhenius plot of the singlet decay rate vs temperature for (a) Rub and (b) *t*BRub polycrystalline films. Decay rates obtained by global analysis of TA data are noted in black squares; prompt and delayed decay rates obtained by global analysis of TRPL data are noted by red circles and blue triangles, respectively. The exponential fits with corresponding activation energies (*E*_a_) are indicated.

To obtain the activation energies for triplet separation in both Rub and *t*BRub films, we performed Arrhenius fits of the lifetimes obtained from TRPL and TA fits. The Arrhenius plots depicting fitted decay rates (*k* = 1/τ) versus inverse temperature are shown in Figure 4. The Arrhenius law implies that the rate depends exponentially on activation energy^26^
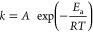
2where *A* is the pre-exponential factor, *E*_a_ is the activation energy, and *R* is the molar gas constant. Interestingly, the Arrhenius plot of the rates recorded for Rub polycrystalline films showed two distinct regions with different activation energies (Figure 4a). The first region with *E*_a_ ≈ 60 meV in the 300–150 K temperature range corresponds to endothermic SF. Similar activation energies in the range of 35–50 meV for Rub crystals were also reported by others.^26,31,47^ Interestingly, at temperatures below 150 K a change of activation energy (*E*_a_ ≈ 15 meV) was observed. The change of activation barrier could be related to the abrupt structural phase transition occurring at a similar temperature range in orthorhombic Rub crystals that leads to slippage in the *b* crystallographic direction.^60^ The change of molecular packing should influence the overlap of π-systems and, thus, the alignment of energy levels. No such change was recorded for *t*BRub polycrystalline films (Figure 4b), which could be related to larger intermolecular distances in the crystal. For *t*BRub negligible negative activation energy (*E*_a_ = −6 meV) of prompt PL component and TA, decay rates were obtained throughout the 77–300 K temperature range. Negative activation energy could be related to suppressed thermal motion and slightly larger exciton delocalization at low temperature that promotes triplet separation.

The Arrhenius fit of the delayed PL component resulted in positive activation energy of 18 and 6 meV for Rub and *t*BRub films, respectively. We suggest that this activation energy is likely related to thermal energy that is required to fuse two separated triplets into a correlated triplet pair. If we consider that delayed nanosecond emission arises from the ^1^(T···T) → ^1^(TT) process, the geminate recombination rate is reflected in the initial decay rate of delayed PL. It must be noted that the decay rates of the delayed PL component shown in Figure 4 were comparable for both crystalline *t*BRub and Rub films, suggesting similar rates of geminate recombination. This implies that the geminate recombination process is less sensitive to the changes in intermolecular coupling compared to triplet separation.

## Conclusions

In summary, we have employed TRPL and TA techniques to study SF and TTA processes as a function of intermolecular coupling and temperature in Rub-based polycrystalline films. A three-step kinetic model [S_0_ + S_1_ → ^1^(TT) ↔ ^1^(T···T) ↔ T_1_ + T_1_] was used to explain exciton fission and recombination processes. We suggest that a distinct delayed ^1^(TT) emission signal appears following the recombination of separated triplet pairs ^1^(T···T). Low thermal activation barriers (6–18 meV) and similar rates for geminate recombination were determined in both crystalline Rub and *t*BRub despite large differences in molecular packing. In contrast, a higher rate of triplet pair separation [^1^(TT) ↔ ^1^(T···T)] in crystalline Rub compared to *t*BRub at room temperature was related to a smaller intermolecular distance and higher triplet energy transport rate. Temperature-activated triplet separation in Rub films was associated with barrier appearing due to lower S_1_ energy with respect to the energy of two triplets 2 × T_1_. Meanwhile, temperature-independent triplet separation (and thus SF) in *t*BRub polycrystalline films was related to the increase of S_1_ energy due to changes in molecular packing in crystal. From the perspective of an efficient TTA material, *t*BRub modification is superior to Rub due to suppressed triplet separation at room temperature, efficient geminate recombination, and temperature stability.

## Supporting Information Available

Crystallographic data of XRD analysis; comparison of simulated and measured powder XRD structures; 2D intensity maps of TRPL data; global analysis of TRPL data; comparison of nanosecond-to-microsecond PL transients; and global analysis of TA data (PDF)
