# Supplementary material for: Sweet Spot of Intermolecular Coupling in Crystalline Rubrene: Intermolecular Separation to Minimize Singlet Fission and Retain Triplet–Triplet Annihilation
Source: J Phys Chem C Nanomater Interfaces. 2022 Aug 30;126(36):15327–35. doi: 10.1021/acs.jpcc.2c04572 (PMC9484276; doi:10.1021/acs.jpcc.2c04572)
Supplement: Supplementary file 1 — jp2c04572_si_001.pdf [file jp2c04572_si_001.pdf]

Supporting information

Sweet-Spot of Intermolecular Coupling in Crystalline Rubrene: Intermolecular Separation to Minimize Singlet Fission and Retain Triplet-Triplet Annihilation

P. Baronas,*[1] G. Kreiza,[1] L. Naimovičius,[1] E. Radiunas,[1] K. Kazlauskas,[1] E. Orentas[2] and S. Juršėnas[1]

[1]Institute of Photonics and Nanotechnology, Vilnius University, Sauletekio 3, LT-10257 Vilnius, Lithuania

[2]Institute of Chemistry, Faculty of Chemistry and Geosciences, Vilnius University, Naugarduko 24, LT-03225 Vilnius, Lithuania.

Contact e-mail: paulius.baronas@ff.vu.lt

1. Crystal structure

Table S1. Crystallographic data and experimental details.

	**Rubrene**	***t*BRub**
empirical formula	$C_{42}H_{28}$	C_{58} H_{60}
Formula weight	532.68	757.10
Temperature (K)	300	300
Crystal system	orthorhombic	monoclinic
Space group	Cmca	P1
a (Å)	26.9065(11)	9.58280(10)
b (Å)	7.1891(2)	10.6008(2)
c (Å)	14.4511(5)	11.5434(2)
Alpha(deg)	90	71.2690(10)
Beta(deg)	90	88.0270(10)
Gamma(deg)	90	89.3040(10)
Volume(Å^3)	2795.33	1109.87
Z	4	1

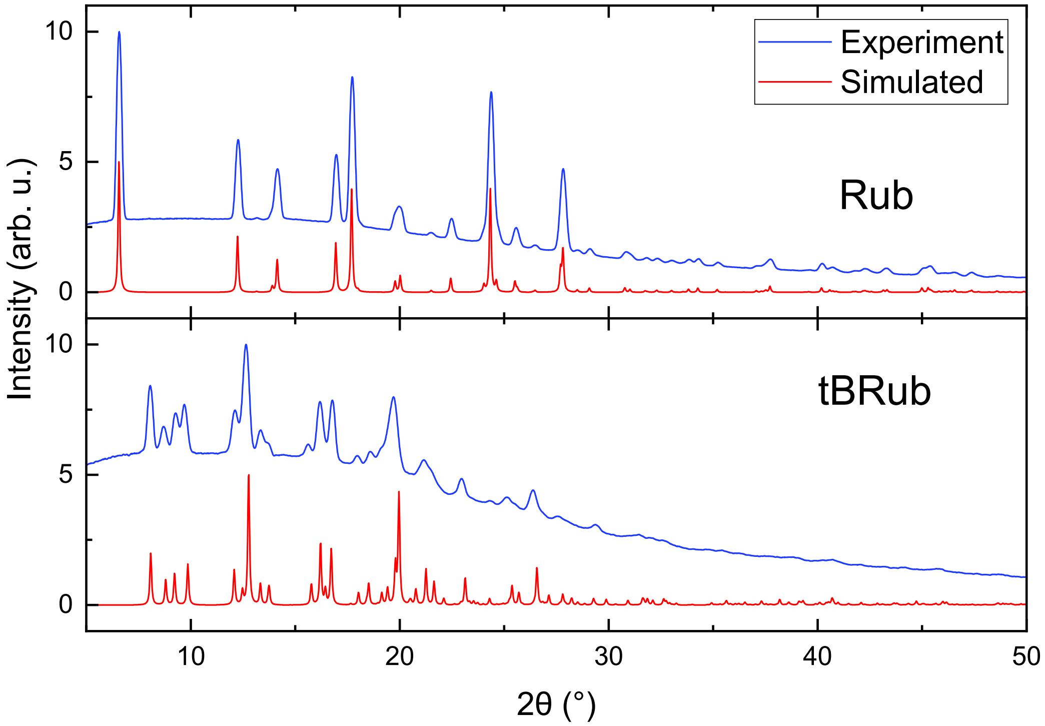

Figure S1. Comparison of powder XRD patterns of samples obtained from investigated polycrystalline Rub and tBRub films with those simulated from single crystal XRD structures.

2. Global analysis of TRPL data

Measured time-resolved photoluminescence (TRPL) of bot Rub and *t*BRub polycrystalline films showed significant spectral red-shifts within first nanoseconds (Figure S2). At low temperatures, these shifts could be identified as decay and rise of two distinctive PL spectra. Therefore, global analysis of TRPL spectra was performed using simple sequential model of 2 states that correspond to prompt and delayed emission. At 77 K temperature the decay associated spectra of prompt and delayed emission (see Figures 3 and 4 in the main text) peaked at 566 nm and 583 nm for rubrene polycrystalline film and 530 nm and 545 nm for *t*BRub polycrystalline film, respectively. In Figure S3a the risetime observed at 583 nm signifies direct population of delayed emission via decay of prompt emission, thus justifying the sequential model. The fitted curves agree well with TRPL data at low temperatures for both films. Global fits using sequential 2 state model were as not effective when fitting longer lived emission tail TRPL data of rubrene films at 300 K. However, for the purpose of extracting singlet fission rates, the fits of the initial parts of the decay were relevant.

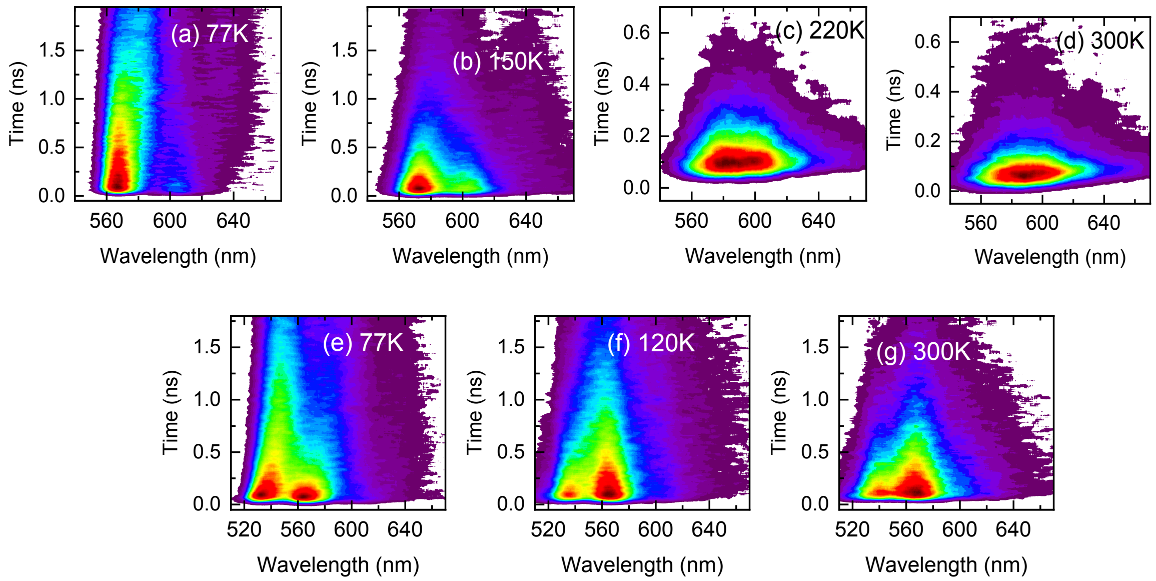

Figure S2. Time-resolved PL intensity plotted as 2D maps of rubrene (a-d) and tBRub (e-f) polycrystalline films measured at different temperatures.

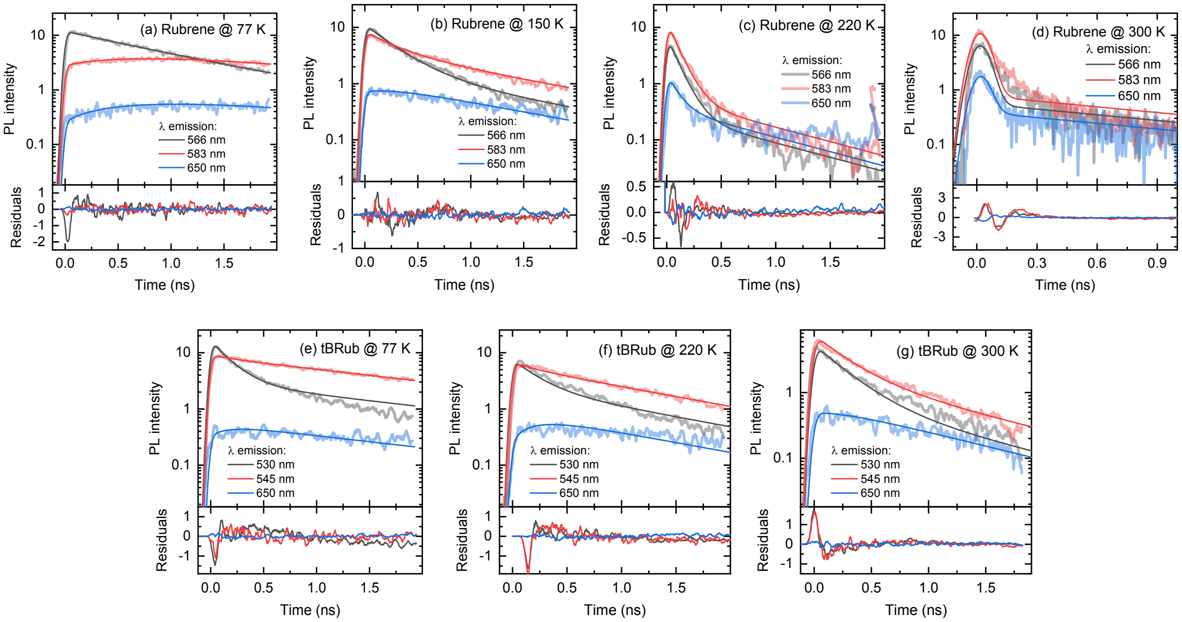

Figure S3. PL transients and corresponding fits at specified emission wavelength of (a-d) Rub and (e-g) *t*BRub polycrystalline films measured in 77-300 K temperature range. Residuals of the fits are presented below the graphs.

3. Nanosecond to microsecond PL transients

The delayed PL transients were normalized at 10 ns after excitation, which should exclude the contribution from nanosecond decay of prompt and delayed PL components. The triplet state of non-interacting monomers of Rub and *t*BRub decays with 100 μs lifetime, which was taken from ref. 22 in the main text.

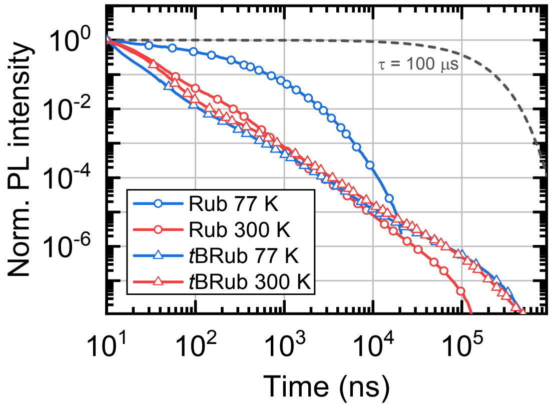

Figure S4. Normalized spectrally integrated PL transients for Rub and *t*BRub polycrystalline films at 77 K and 300 K. Single-exponential decay curve (dashed line) with 100 μs lifetime serves as a reference for decay of triplet state in Rub and *t*BRub monomers.

4. Global analysis of TA data

Similarly to global analysis of TRPL data, sequential model with 2 states was used for transient absorption data. However, the 2 states corresponded to singlets and triplets with distinct spectral features in the transient absorption spectra. Decay associated spectra and transients with corresponding fits of rubrene and tBRub films is presented in Figures S5 and S6, respectively.

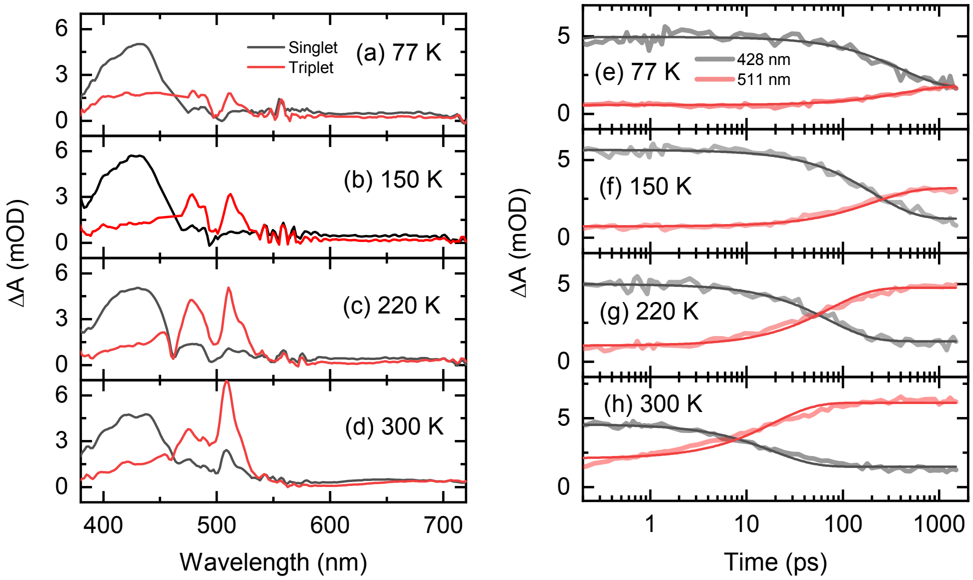

Figure S5. Global analysis of Rub polycrystalline film temperature dependent transient absorption spectra. (a-d) decay associated spectra of singlet (black line) and triplet (red line) states. (e-h) transients at 428 nm (black line) and 511 nm (red line) and corresponding fits.

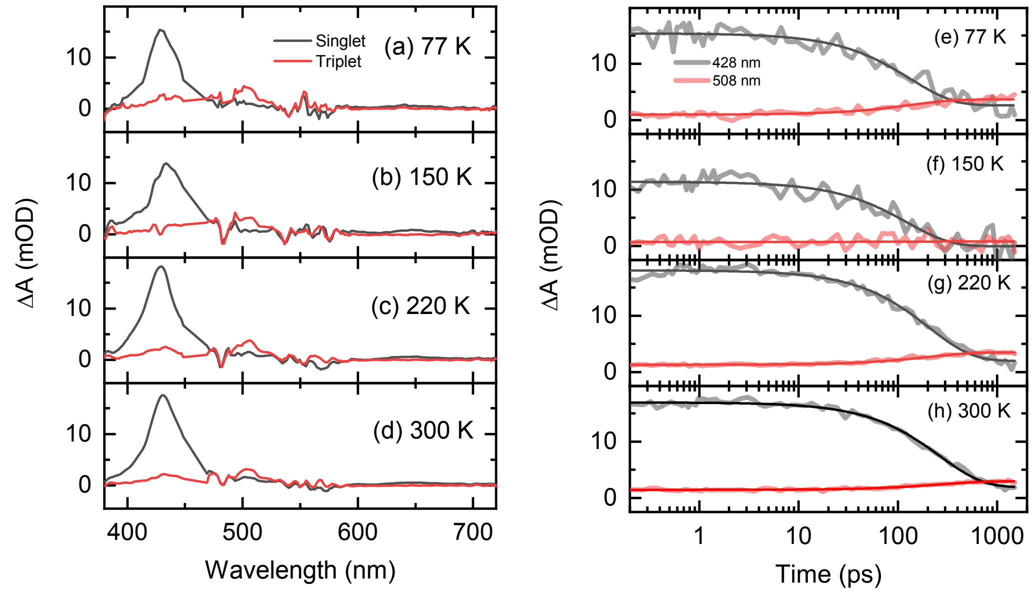

Figure S6. Global analysis of *t*BRub polycrystalline film temperature dependent transient absorption spectra. (a-d) decay associated spectra of singlet (black line) and triplet (red line) states. (e-h) transients at 428 nm (black line) and 508 nm (red line) and corresponding fits.
